# Preliminary Prognostication for Good Neurological Outcomes in the Early Stage of Post-Cardiac Arrest Care

**DOI:** 10.3390/diagnostics13132174

**Published:** 2023-06-26

**Authors:** Sunghyuk Lee, Jung Soo Park, Yeonho You, Jin Hong Min, Wonjoon Jeong, Hong Joon Ahn, Yong Nam In, Yong Chul Cho, In Ho Lee, Jae Kwang Lee, Changshin Kang

**Affiliations:** 1 Department of Emergency Medicine, Chungnam National University Hospital, 282 Munhwa-ro, Jung-gu, Daejeon 35015, Republic of Korea; rococo159@cnuh.co.kr (S.L.); cpcr@cnu.ac.kr (J.S.P.); yyo1003@naver.com (Y.Y.); gardenjun@hanmail.net (W.J.); jooniahn@daum.net (H.J.A.); boxter73@naver.com (Y.C.C.); 2 Department of Emergency Medicine, College of Medicine, Chungnam National University, 282 Mokdong-ro, Jung-gu, Daejeon 35015, Republic of Korea; laphir2006@naver.com (J.H.M.); ynsoft@naver.com (Y.N.I.); 3 Department of Emergency Medicine, Chungnam National University Sejong Hospital, 20, Bodeum 7-ro, Sejong 30099, Republic of Korea; 4 Department of Radiology, Chungnam National University Hospital, 282 Munhwa-ro, Jung-gu, Daejeon 35015, Republic of Korea; leeinho1974@hanmail.net; 5 Department of Radiology, College of Medicine, Chungnam National University, 282 Mokdong-ro, Jung-gu, Daejeon 35015, Republic of Korea; 6 Department of Emergency Medicine, Konyang University Hospital, College of Medicine, Daejeon 35365, Republic of Korea; neokey@naver.com

**Keywords:** out-of-hospital cardiac arrest, prognosis, clinical examination, computed tomography, neuron specific enolase

## Abstract

We investigated prognostic strategies for predicting good outcomes in the early stage of post-cardiac-arrest care using multiple prognostic tests that are available until 24 h after the return of spontaneous circulation (ROSC). A retrospective analysis was conducted on 138 out-of-hospital cardiac-arrest patients who underwent prognostic tests, including the gray–white-matter ratio (GWR-BG), the Glasgow Coma Scale motor (GCS-M) score before sedative administration, and the neuron-specific enolase (NSE) level measured at 24 h after the ROSC. We investigated the prognostic performances of the tests as single predictors and in various combination strategies. Classification and regression-tree analysis were used to provide a reliable model for the risk stratification. Out of all the patients, 55 (44.0%) had good outcomes. The NSE level showed the highest prognostic performance as a single prognostic test and provided improved specificities (>70%) and sensitivities (>98%) when used in combination strategies. Low NSE levels (≤32.1 ng/mL) and high GCS-M (≥4) scores identified good outcomes without misclassification. The overall accuracy for good outcomes was 81.8%. In comatose patients with low NSE levels or high GCS-M scores, the premature withdrawal of life-sustaining therapy should be avoided, thereby complying with the formal prognostication-strategy algorithm after at least 72 h from the ROSC.

## 1. Introduction

In the last few decades, the development of post-cardiac-arrest-care bundles such as targeted temperature management (TTM) and hemodynamic optimization have led to improved outcomes, i.e., patients discharged from hospital with meaningful neurological recovery [1,2,3]. Despite these advanced efforts, survival rates with good neurological outcomes are reportedly dismally low [4,5,6]. Several studies have reported that the withdrawal of life-sustaining therapy because of perceived poor neurological outcome (WLST-N) is highly associated with attributable mortality [7,8,9,10].

Although the current guidelines recommend deferring neurological prognostication until at least 72 h after the return of spontaneous circulation (ROSC) [11,12], the incidence of early WLST-N (i.e., within 72 h of the ROSC) is reportedly considerably high [13]. A recent study reported that the confounding effects of sedation and the insufficiency of a multimodal approach to prognostication might be attributed to inappropriate early WLST-N in patients with out-of-hospital cardiac arrest (OHCA) [7,14].

Therefore, this study aimed to (1) determine the prognostic performances of prognostic tests that are available until 24 h from the ROSC and free from the sedation effect, (2) consider the practical implications of investigations for reliable prognostication in patients with post-cardiac-arrest care, and (3) suggest the need for preliminary prognostication to reduce the inappropriate WLST-N in the early stage of post-cardiac-arrest care for OHCA.

## 2. Materials and Methods

### 2.1. Study Design and Population

This was a single-center, retrospective, observational, and registry-based study. We prospectively collected data on patients who received post-cardiac arrest care after OHCA between May 2018 and August 2022 from the registry at a tertiary-care hospital (Chungnam National University Hospital (CNUH), Daejeon, Korea). The Institutional Review Board approved the study protocol before data collection (CNUH-2022-11-041). This study included adult (>18 years) patients who sequentially underwent multiple prognostic tests within 24 h of post-cardiac-arrest care after OHCA, as follows: (1) brain computed tomography (CT), (2) Glasgow Coma Scale motor (GCS-M) score before administration of sedatives or paralytics, and (3) neuron-specific enolase (NSE) level at 24 h after the ROSC. Among these patients, those who underwent extracorporeal membrane oxygenation (ECMO) and died due to cardiac death during post-cardiac-arrest care (i.e., 72–96 h after the ROSC) were excluded from this study.

### 2.2. Post-Cardiac-Arrest Care

Patients who had a GCS-M score of <6 after the ROSC underwent post-cardiac arrest care. The TTM was performed using an external cooling device (Arctic Sun^®^ 5000; BD, Franklin Lakes, NJ, USA). The targeted temperature of 33 °C or 36 °C was maintained for 24 h with rewarming to 37 °C at a rate of 0.25 °C per hour, and it was monitored using an esophageal or bladder-temperature probe. Sedatives (midazolam) and paralytics (cisatracurium or rocuronium) were administered. If there was evidence of electrographic seizure or a clinical diagnosis of seizure, anti-epileptic drugs (lorazepam, levetiracetam, and/or valproate) were administered. All patients received standard intensive care according to our institutional intensive care unit’s protocol, based on the 2021 international guidelines for post-cardiac-arrest care [11]. Early WLST-N was not performed in this study (Supplementary File).

### 2.3. Data Acquisition

#### 2.3.1. Baseline Characteristics

The following variables were extracted from the data registry: age; sex; Charlson comorbidity index; sequential organ-failure-assessment score within the first 24 h after admission; witnessed collapse; bystander cardiopulmonary resuscitation (CPR); time from CPR to the ROSC (low-flow time); first monitored rhythm; etiology of cardiac arrest; time to the targeted temperature; performance of the brain CT; and measurement of GCS from the ROSC.

#### 2.3.2. Gray-White Matter Ratio

Brain CT was performed before the administration of TTM. It was obtained in 5 mm slices using a 64-channel system (Somatom Sensation 64, Siemens Healthineers, Erlangen, Germany). Two board-certified neuroradiologists, who were blinded to clinical outcomes, measured the Hounsfield units (HU) of the putamen (P), caudate nucleus (CN), posterior limb of the internal capsule (PIC), and corpus callosum (CC) at the basal ganglia level. Circular regions of measurement (9–11 mm^2^) were manually placed over these anatomical regions, and the average attenuation in HU was recorded. The gray–white-matter ratio at the basal ganglia level (GWR-BG) was calculated according to a previously reported equation: GWR-BG = (HU_P_ + HU_CN_)/(HU_PIC_ + HU_CC_) (Supplementary Figure S1) [15]. The average GWR-BG between the two reviewers was finally used in this analysis. Inter-rater reliabilities of GWR-BG were determined by two experts. (Supplementary Method and Table S1).

#### 2.3.3. Clinical Examination

The GCS-M score was extracted from the electronic medical records. A trained nurse in the emergency room measured the GCS-M once every 1 h after the ROSC or with any change in mental status. Among these recorded serial GCS-M scores, the highest score determined before the administration of any sedative or paralytic was used in this analysis to exclude confounding effects from sedation (Supplementary Figure S2). The time required to determine the GCS-M scores used in this study was also investigated.

#### 2.3.4. Serum Biomarkers

The data on brain-specific biomarkers, NSE level, at 24 h after the ROSC were used for this analysis. All serum samples were obtained from an arterial catheter and analyzed in one laboratory, namely the Green Cross Laboratory (GC Labs; Yongin, Gyeonggi-do, Korea). The NSE concentration was determined using an electrochemiluminescence immunoassay with Elecsys NSE^®^ (COBAS e801; Roche Diagnostics, Basel, Switzerland). The measurement range of the NSE level was 0.1–300 ng/mL. Finally, GC Labs was compliant with the relevant national and international guidelines.

### 2.4. Outcome

Neurological outcomes were assessed 3 months after OHCA using the cerebral performance category (CPC) score (Supplementary File). The primary outcome in this study was a good neurological outcome, defined as a CPC score of 1 or 2.

### 2.5. Statistical Analysis

Categorical and continuous variables were presented as counts with percentiles and median values with interquartile ranges (IQRs), respectively. Categorical variables were compared between groups using Chi-square or Fisher’s exact tests, as appropriate. Continuous variables were compared between groups using the Mann–Whitney U test. Receiver operating characteristic (ROC) curves were generated for each predictor, and probability values of combination models were calculated using binary logistic regression analysis. Subsequently, the predictive performance was determined using the area under the ROC curve, specificity, and sensitivity with a 95% confidence interval (CI). The specificity for a good neurological outcome was demonstrated as the highest value under a sensitivity of >98% to guarantee a low false pessimistic prediction, potentially leading to an inappropriate WLST-N. Classification and regression tree (CART) analysis is a non-parametric decision-tree technique that can be used to provide a simple and reliable model for risk stratification [16]. This technique has been used in a variety of clinical research settings to predict clinical outcomes [17]. In this study, CART analysis was applied to predict good neurological outcomes in relation to prognostic tests.

Statistical analysis was performed using SPSS 26.0 for windows (IBM Corp., Armonk, NY, USA) and MedCalc 15.2.2 (MedCalc Software Ltd., Ostend, Belgium). The significance level was set at *p* < 0.05.

## 3. Results

### 3.1. Baseline Characteristics of Total Cohort

**Figure 1:**
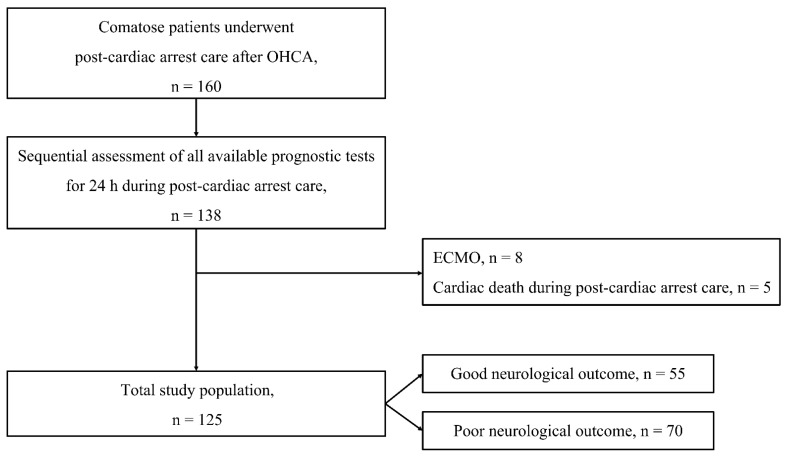
Flow diagram of included patients. Abbreviations: OHCA, out-of-hospital cardiac arrest; ECMO, extracorporeal membrane oxygenation.

Of the 160 patients who underwent post-cardiac-arrest care after OHCA, 138 underwent all the available prognostic tests for the first 24 h during post-cardiac arrest care. Of these, 13 (eight (ECMO) and five (cardiac death during post-cardiac arrest care) patients were excluded from this study (Figure 1). Of the remaining 125 patients, 55 (44.0%) and 70 (56.0%) had good and poor neurological outcomes, respectively.

Several cardiac-arrest characteristics, including witnessed arrest, bystander CPR, shockable rhythm, cardiac etiology, low-flow time, and sequential organ-failure-assessment score, showed significant differences between the good- and poor-neurological-outcome groups (*p* < 0.01; Table 1). In addition, the time to measure the highest GCS-M score from the ROSC was longer in the good- versus the poor-neurological-outcome group (2.3 h (IQR, 1.1–3.2) vs. 3.6 h (IQR, 2.2–6.0), *p* = 0.003; Table 1).

### 3.2. Associations between Prognostic Tests and Neurological Outcomes

The GWR-BG and GCS-M scores were significantly higher in the good-neurological-outcome group than those in the poor-neurological-outcome group (GWR-BG: 1.25 (IQR, 1.20–1.30) vs. 1.16 (IQR, 1.05–1.22), *p* < 0.001; GCS-M score: 4 (IQR, 1–4) vs. 1 (IQR, 1–1), *p* < 0.001; Table 2). The NSE levels were significantly lower in the good-neurological-outcome group than in the poor-neurological-outcome group (24.3 ng/mL (IQR, 18.7–31.0) vs. 83.9 ng/mL (IQR, 37.7–251.3), *p* < 0.001; Table 2).

### 3.3. Prognostic Performances of Single Prognostic Tests and Combination Strategies

The GWR-BG and NSE levels achieved a sensitivity of 100%, whereas the GCS-M did not produce specificity, with a sensitivity of >98% (Table 3). The NSE levels showed relatively high specificity and higher sensitivity than the GWR-BG (Table 3). The combination strategies including NSE level (i.e., GWR-BG + NSE, GCS-M + NSE, and all the prognostic tests) all showed a specificity of >70% with a sensitivity of >98% (Table 4), whereas a combination of the GWR and GCS-M scores did not show improved specificity (37.1% (95% CI, 25.9–49.5); Table 4). In the nested analysis of the combination strategies, a combination strategy using all the prognostic tests demonstrated significantly higher prognostic performance compared with the other combination strategies used, i.e., GWR-BG + GCS-M (*p* = 0.002; Table 4) and GWR-BG + NSE (*p* = 0.005; Table 4). The combination strategy of GCS-M score and NSE level showed comparable specificity to a combination of all the prognostic tests (*p* = 0.09; Table 4).

### 3.4. CART Analysis for Good Neurological Outcomes

**Figure 2:**
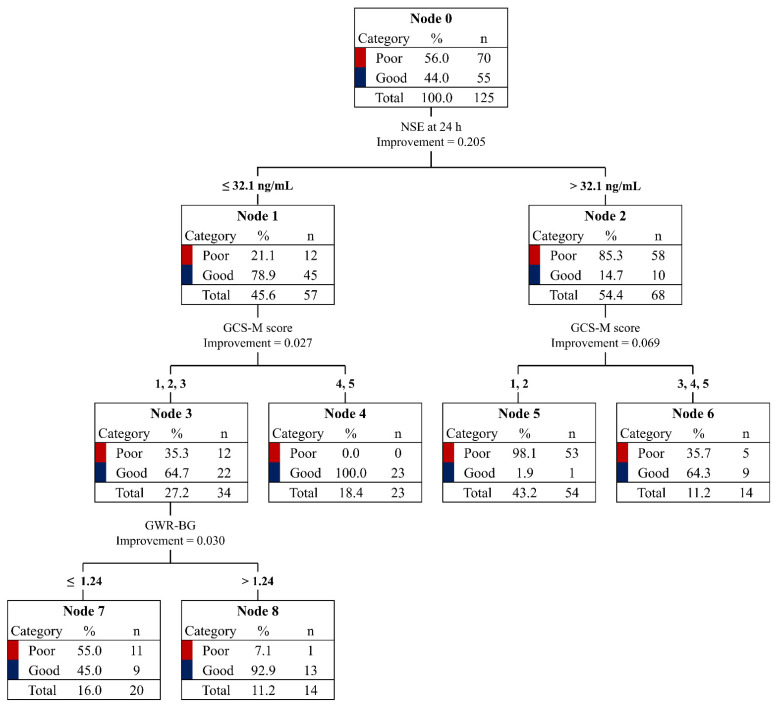
Classification and regression-tree analysis for prediction of good and poor neurological outcomes. Abbreviations: NSE, neuro-specific enolase; GCS-M, Glasgow Coma Scale motor; GWR-BG, gray–white-matter ratio at the basal ganglia level.

Figure 2 shows the decision tree for good neurological outcomes in the cohort. The NSE levels at 24 h contributed the most significantly to this decision tree (improvement = 0.21), whereas the GWR-BG made little contribution to the identification of patients with good neurological outcomes (improvement = 0.03). The CART analysis identified three prognostic groups for patients with NSE levels ≤ 32.1 ng/mL (Node 1) and two prognostic groups for those with NSE levels > 32.1 ng/mL (Node 2). In the low-NSE cohort, GCS-M scores of ≥4 identified patients with good neurological outcomes without any false-positive results (Node 4), whereas GCS-M scores of ≤3 required the addition of the GWR-BG to finally identify the outcome. A GWR-BG of >1.24 identified 13 good neurological outcomes in the cohorts with NSE levels of ≤32 ng/mL and GCS scores of ≤3 (Node 8). In the high-NSE cohort, GCS-M scores of 1 and 2 identified 53 negative results with a sensitivity of >98% (Node 5). The accuracies of the identification of good and poor neurological outcomes were 81.8% and 91.4%, respectively, and the total accuracy was 87.2%.

## 4. Discussion

Because prognostic tests performed in the early stages might be confounded by the effect of sedation and have the potential for misclassification, high-quality evidence supporting the application of early prognostication in clinical practice has not been found [18]. To account for these major hurdles for early prognostication for patients with post-cardiac-arrest care after OHCA, this study evaluated the prognostic performances of selective prognostic tests, which were obtained within 24 h of or at 24 h after the ROSC, with patients free of sedation effects, and investigated their combination strategies in a non-WLST setting. Our findings showed that the combination strategies were associated with improved prognostic performance compared with that of each prognostic test alone. This emphasizes that the multimodal approach is more appropriate for prognostication than single prognostic tests, even if prognostication is performed in the early stage and aimed at good neurological outcomes [19]. In addition, a prognostic algorithm with a specific cut-off value for each test was demonstrated using CART analysis to identify the prognostic group in comatose patients at 24 h post-cardiac-arrest care, with an overall accuracy of 87.2%. Notably, all 23 patients with NSE levels ≤ 32.1 ng/mL and GCS-M scores of 4 or 5 showed good neurological outcomes (Node 4). This finding suggests that the NSE level obtained at 24 h and the highest GCS-M score measured before sedation may help identify whether a comatose patient has a chance of achieving a neurologically meaningful recovery in the early stage of post-cardiac-arrest care for OHCA.

Because of the premature discriminative power to accurately form prognoses, the current guideline states that the decision of WLST should be postponed to at least 72 h after cardiac arrest [11,20]. Given the advantages of predicting good neurological outcomes suggested by Sandroni et al. [21] and the fact that only 8.8% of early WLSTs result in cardiac death [7], the detection of a chance of good neurological recovery in the early stage of post-cardiac arrest care can not only reassure clinicians and patients’ caregivers, but also help in the decision regarding the allocation of limited medical resources to those who are likely to benefit from advanced care. Thus, we suggest that early prognostication should focus on good rather than poor neurological outcomes. Although preliminary prognostication performed within 72 h would have several limitations compared with the formal prognostication suggested by the guidelines, it may lead to reduced premature and inappropriate WLST-N, or at least have a role in postponing WLST-N decisions until 72 h after the ROSC in patients who have a chance of recovery. Notably, the accuracy of our algorithm is insufficient for application in clinical practice; thus, our findings and suggestions may be considered among the first steps in the development of a preliminary prognostication method to avoid inappropriate and premature WLST-N.

The cut-off value for the identification of a prognostic subgroup with NSE was 32 ng/mL, with a specificity of 82.9% and a sensitivity of 81.8% in this study, whereas a previous study suggested a cut-off value of NSE as a normal value of 17–18 ng/mL [22,23]. In addition, the NSE cut-off value with which we achieved a sensitivity of 100% was also higher than that of a previous study (85.3 ng/mL vs. 75.0 ng/mL) [24]. We suggest that this higher cut-off value of NSE might have resulted from the heterogeneity of cardiac-arrest characteristics. Our study population showed lower rates of shockable rhythm and witnessed cardiac arrest than those reported in previous studies [22,25]. This may significantly affect the baseline NSE value, leading to a discrepancy in the NSE cut-off value. Nevertheless, the NSE levels demonstrated similar sensitivity (81.8% vs. 89.9%) [23] and a positive predictive value (61.8% vs. 63.1%) [24], and were associated with improved prognostic performance for good neurological outcomes through a combination strategy. Hence, we suggest that the measurement of the NSE level can be of great clinical utility as an early prognostic test for good neurological outcomes.

To exclude the confounding effect of sedation, we used the GCS-M scores and GWR-BG results measured in the extremely early stages (at medians of 2.5 h and 1.4 h after the ROSC, respectively) as the prognostic tests in this analysis. The accuracy of these tests in the prediction of good neurological outcomes was not particularly high, and the combination strategies were not associated with improvements in prognostic performance; instead, the specificity with a sensitivity of >98% was lower than that of GWR-BG alone (37.1% vs. 38.6%). These results suggest that the use of these tests as early prognostic markers for the prediction of good neurological outcomes can increase redundancy rather than prognostic performance. The GCS-M score showed a higher contribution to establishing the decision tree in the CART analysis than the GWR-BG in the low-NSE prognostic group (improvement of 0.027 vs. 0.030). In addition, no contributions from the GWR-BG were observed in the high-NSE-level prognostic group. The limitation of the GWR-BG on brain CT obtained early (an average of 2 h after the ROSC) was described in several previous studies [26,27,28]. Therefore, our result regarding the limitation of the GWR-BG measured at an early stage is in line with the paucity of evidence regarding the prediction of good neurological outcomes using brain CT [21]. In contrast, the GCS-M scores measured in patients without sedation effects partly contributed to the identification of good and poor neurological outcomes; 23 (Node 4) and 53 (Node 5) patients were identified using a specific cut-off value in the prognostic groups with low (≤32.1 ng/mL) and high (>32.1 ng/mL) NSE, respectively. Moreover, previous multicenter studies have reported that high GCS-M scores yielded specificity values of 98% and 84%, respectively, for good neurological outcomes [2,25]. In addition, a recent systemic review suggested GCS-M scores measured in patients without sedation as one of the indices for predicting good neurological outcomes, while the GWR-BG was not included in these indices [21]. Hence, it seems appropriate to use the GCS-M score as an index of a multimodal approach for prognostication rather than the GWR-BG, if it is measured without the confounding effect of sedation.

This study had several limitations. Most notably, this study was conducted retrospectively, in a single center, with a small sample size. The reliance on the OHCA registry in our institution and medical records inevitably led to potential bias. Therefore, a further prospective multicenter investigation with a large sample size is necessary. Not all of the 160 patients who underwent post-cardiac-arrest care after OHCA was deemed necessary could be analyzed, as data from 22 patients could not be included in this study due to the insufficiency of the prognostic tests performed at 24 h after the ROSC. This selection bias may have affected our results considerably. Moreover, although the neurological outcomes measured at 6 months after the ROSC ere widely accepted in previous studies on post-cardiac-arrest care, they were not investigated in this study, since the use of long-term outcomes in the analysis could have led to differences in the prognostic performances measured in this study. Finally, we used a simple quantitative analysis of the brain CT scans. Although another quantitative analysis on brain CT, such as the Alberta Stroke Program Early CT Score–Bilateral has been suggested as a predictor with which to supplement some of the limitations of the GWR-BG [29,30], it is not commonly performed in clinical practice and essentially needs specific software. Despite the known limitations of GWR-BG, we used this simple method as a prognostic test for a multi-modal approach because it may be more efficient in clinical practice.

## 5. Conclusions

Low NSE levels obtained at 24 h and high GCS-M scores observed before sedation were strongly associated with improved prognostic performance for early prognostication aimed at good neurological outcomes, which may help to identify whether a comatose patient has a chance of achieving a neurologically meaningful recovery after post-cardiac arrest care. Thus, in comatose patients with low NSE levels or high GCS-M scores, premature WLST should be avoided, thereby complying with the formal prognostication-strategy algorithm at least 72 h after the ROSC. Further multi-center studies with large sample sizes are required to confirm our results.

## Figures and Tables

**Table 1:** Baseline demographics and characteristics.

Variables	Total Patients n = 125	Good Neurological Outcome n = 55	Poor Neurological Outcome n = 70	*p*
Age, years	58 (42–70)	59 (45–70)	58 (40–69)	0.97
Sex, male	93 (74.4)	45 (81.8)	48 (68.6)	0.11
Charlson comorbidity index	2.0 (1.0–4.0)	2.5 (1.0–4.0)	2.0 (0.0–4.0)	0.61
Hypertension	42 (33.6)	18 (32.7)	24 (34.3)	0.86
Diabetes mellitus	37 (29.6)	15 (27.3)	22 (31.5)	0.82
Myocardial infarction	13 (10.4)	8 (14.5)	5 (7.1)	0.18
Cerebrovascular attack	4 (3.2)	2 (3.6)	2 (2.9)	0.81
Lung disease	7 (5.6)	1 (1.8)	6 (8.6)	0.10
Renal disease	19 (15.2)	8 (14.5)	11 (15.8)	0.92
Liver disease	4 (3.2)	2 (3.6)	2 (2.8)	0.49
Malignancy	7 (5.6)	4 (7.3)	3 (4.3)	0.50
Cardiac arrest characteristics				
Witnessed	75 (60.0)	48 (87.3)	27 (38.6)	<0.001
Bystander CPR	86 (68.8)	45 (81.8)	41 (58.6)	0.005
Shockable rhythm	40 (32.0)	33 (60.0)	7 (10.0)	<0.001
Cardiac etiology	54 (43.2)	39 (70.9)	15 (21.4)	<0.001
No-flow time, min	1.0 (0.0–13.0)	1.0 (0.0–2.0)	9.0 (0.0–23.0)	<0.001
Low-flow time, min	20.0 (10.3–30.8)	13.0 (8.0–19.0)	28.0 (19.8–37.3)	<0.001
SOFA score	10 (8–12)	8 (7–11)	11 (9–12)	0.62
Time (from the ROSC), hours				
to targeted temperature	5.8 (4.6–7.3)	5.6 (4.5–7.0)	5.9(4.6–7.5)	0.88
to perform brain CT	1.4 (0.9–2.6)	1.1 (0.8–2.1)	1.7 (0.9–2.9)	0.22
to measure GCS-M score	2.5 (1.5–4.2)	2.3 (1.1–3.2)	3.6 (2.2–6.0)	0.003
CAG performed	43 (34.4)	35 (63.6)	8 (11.4)	<0.001
Abnormal finding				<0.001
LM	2 (4.7)	2 (5.7)	0 (0)	
LAD	13 (30.2)	10 (28.6)	3 (37.5)	
LCx	9 (20.9)	7 (20.0)	2 (25.0)	
RCA	10 (23.3)	9 (25.7)	1 (12.5)	

Data are presented as median values (interquartile ranges) or numbers (%). Abbreviations: HTN, hypertension; DM, diabetes mellitus; MI, myocardial infarction; CI, cerebral infarction; CPR, cardiopulmonary resuscitation; SOFA, sequential organ failure assessment; GCS-M, Glasgow Coma Scale motor; ROSC, return of spontaneous circulation; CT, computed tomography; CAG, coronary angiography; LM, left main; LAD, left anterior descending; LCx, left circumflex; RCA, right coronary artery.

**Table 2:** Associations between each prognostic test and neurological outcomes.

Prognostic Tests	Total Patients	Good Neurological Outcome n = 55	Poor Neurological Outcome n = 77	*p*
GWR-BG	1.20 (1.13–1.28)	1.25 (1.20–1.30)	1.16 (1.05–1.22)	<0.001
GCS-M score	1 (1–4)	4 (1–4)	1 (1–1)	<0.001
NSE level, ng/mL	35.8 (22.9–110.0)	24.3 (18.7–31.0)	83.9 (37.7–251.3)	<0.001

Abbreviations: GWR-BG, gray–white-matter ratio at the basal ganglia level; GCS-M, Glasgow Coma Scale motor score; NSE, neuron-specific enolase.

**Table 3:** Prognostic performances of single predictors of good neurological outcomes.

Values	Cut-off	Specificity (95% CI)	Sensitivity (95% CI)	PPV (95% CI)	NPV (95% CI)	TP	FP	TN	FN
GWR-BG	>1.2	70.0 (57.9–80.4)	72.7 (59.0–83.1)	65.6 (52.3–77.3)	76.6 (64.3–86.2)	40	21	49	15
	>1.11	41.4 (29.8–53.8)	98.2 (90.3–100.0)	56.8 (46.3–67.0)	96.7 (82.8–99.9)	54	41	29	1
	>1.09	38.6 (27.2–51.0)	100.0 (93.5–100.0)	56.1 (45.7–66.1)	100 (87.2–100.0)	55	43	27	0
GCS-M score	>2	88.6 (78.7–94.9)	65.6 (51.4–77.8)	81.8 (67.3–91.8)	76.5 (65.8–85.2)	36	8	62	19
NSE level, ng/mL	≤32	82.9 (72.0–90.8)	81.8 (69.1–90.9)	78.9 (66.1–88.6)	85.3 (74.6–92.7)	45	12	58	10
	≤58.9	58.6 (46.2–70.2)	98.2 (90.3–100.0)	65.1 (53.8–75.2)	97.6 (87.4–99.9)	54	29	41	1
	≤85.3	51.4 (39.2–63.6)	100.0 (93.5–100.0)	61.8 (50.9–71.9)	100.0 (90.3–100.0)	55	34	36	0

Abbreviations: CI, confidence interval; PPV, positive predictive value; NPV, negative predictive value; TP, true positive; FP, false positive; TN, true negative; FN, false negative; GWR-BG, gray–white-matter ratio at the basal ganglia level; GCS-M, Glasgow Coma Scale motor; NSE, neuron-specific enolase.

**Table 4:** Sensitivities and false-negative rate of combination strategies for predicting good neurological outcome.

Values	AUC (95% CI)	*p*-Value ^a^			Specificity (95% CI)	Sensitivity (95% CI)	PPV (95% CI)	NPV (95% CI)
GWR-BG + GCS-M score	0.89 (0.82–0.94)	*			37.1 (25.9–49.5)	98.2 (90.3–100.0)	55.1 (44.7–65.2)	96.3 (81.0–99.9)
GWR-BG + NSE level	0.90 (0.83–0.94)	0.85	*		71.4 (59.4–81.6)	98.2 (90.3–100.0)	73.0 (61.4–82.6)	98.0 (89.6–100.0)
GCS-M score + NSE level	0.93 (0.87–0.97)	0.15	0.20	*	72.9 (60.9–82.8)	98.2 (90.3–100.0)	74.0 (62.4–83.5)	98.1 (89.7–100.0)
GWR-BG + GCS-M score + NSE level	0.96 (0.90–0.98)	0.002	0.005	0.09	77.1 (65.6–86.3)	98.2 (90.3–100.0)	77.1 (65.6–86.3)	98.2 (90.3–100.0)

^a^ Statistical difference between AUC value were calculated by using DeLong’s test. * References for a nested analysis for AUC value. Abbreviations: AUC, area under the ROC curve; CI, confidence interval; PPV, positive predictive value; NPV, negative predictive value; GWR-BG, gray–white-matter ratio at the basal ganglia level; GCS-M, Glasgow Coma Scale motor; NSE, neuron-specific enolase.

## Data Availability

The data presented here are available on request from the corresponding author. The data are not publicly available because of ethical concerns.

## Supplementary Materials

The following supporting information can be downloaded at: https://www.mdpi.com/article/10.3390/diagnostics13132174/s1. Figure S1: Brain-CT images showing measurements in Hounsfield units for calculating GWR-BG. Supplementary Methods: Statistical method used in analysis of inter-rater reliability of GWR-BG in brain CT. Figure S2: Scheme for data acquisition of prognostic tests until 24 h after the return of spontaneous circulation during post-cardiac-arrest care. Table S1: Inter-rater reliability analysis of the calculated GWR-BG on brain CT.
