# Supplementary material for: Preliminary Prognostication for Good Neurological Outcomes in the Early Stage of Post-Cardiac Arrest Care
Source: Diagnostics (Basel). 2023 Jun 26;13(13):2174. doi: 10.3390/diagnostics13132174 (PMC10340272; doi:10.3390/diagnostics13132174)
Supplement: Supplementary file 1 [file diagnostics-13-02174-s001.zip › diagnostics-2449619-supplementary.pdf]

Additional File S1

Title: Preliminary prognostication for good neurological outcomes in the early stage of post-cardiac arrest care

Supplementary S1. Withdrawal of life-sustaining therapy in our institution

Figure S1. Brain CT images showing measurements in Hounsfield units for calculating GWR-BG

Supplemental Method. Statistical method in an analysis for inter-rater reliability of GWR-BG on brain CT

Figure S2. Scheme for data acquisition of prognostic tests until 24 h after return of spontaneous circulation during post-cardiac arrest care

Supplementary S2. Cerebral Performance Category score

Table S1. Inter-rater reliability analysis of the calculated GWR-BG on brain CT

Abbreviations: GWR-BG, grey-white matter ratio at basal ganglia level; CT, computed tomography; CPC, Cerebral Performance Category

Supplementary S1. Withdrawal of life-sustaining therapy in our institution

Since 2018, the WLST has been highly restricted in Korea [1]. Legally, it requires declarations for irreversible and unrecoverable status from at least two physicians, even if the family has a strong willingness to do so. Therefore, the physicians in charge of post-cardiac arrest care do not encourage WLST, and it is restrictively performed in patients with brain death who are denied organ donation from a caregiver or family. In addition, Korea adopts a highly strict qualification for brain death, such as a flat (< 2 μV) electroencephalogram for 30 min [2].

Figure S1. Brain computed tomography images showing measurements in Hounsfield units at the basal ganglia level

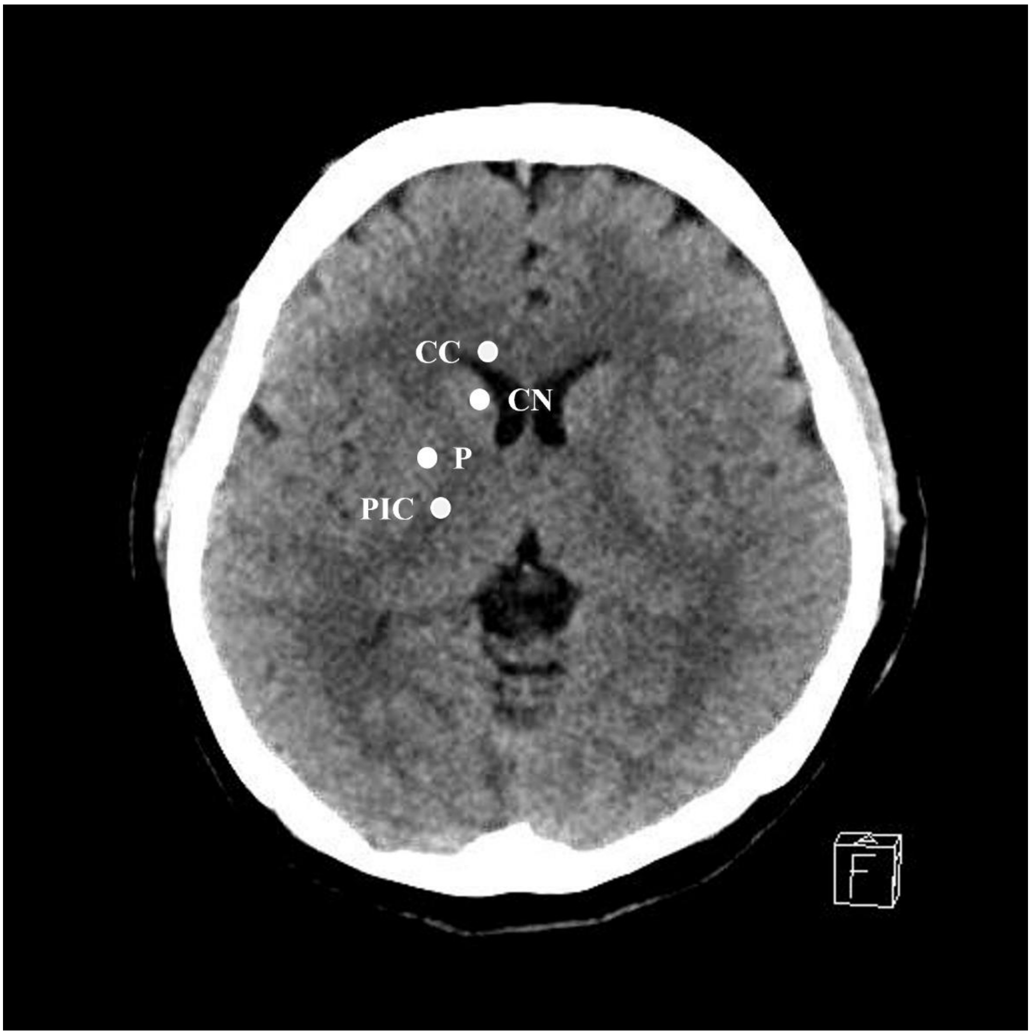

Abbreviations**:** CC, corpus callosum; CN, caudate nucleus; P, putamen; PIC, posterior limb of the internal capsule

Supplemental Method. Statistical method used for analysis of inter-rater reliability of GWR-BG on brain computed tomography

Inter-rater reliability was determined using the intraclass correlation coefficient (ICC), according to the characteristics of the variables. ICC values of <0.4, 0.4–0.75, and >0.75 indicated a poor, fair-to-good, and excellent agreement, respectively [3]

Abbreviations: GWR-BG, grey and white matter ratio at basal ganglia level; CT, computed tomography

Figure S2. Scheme for a protocol for prognostication until 24 h after return of spontaneous circulation in post-cardiac arrest care

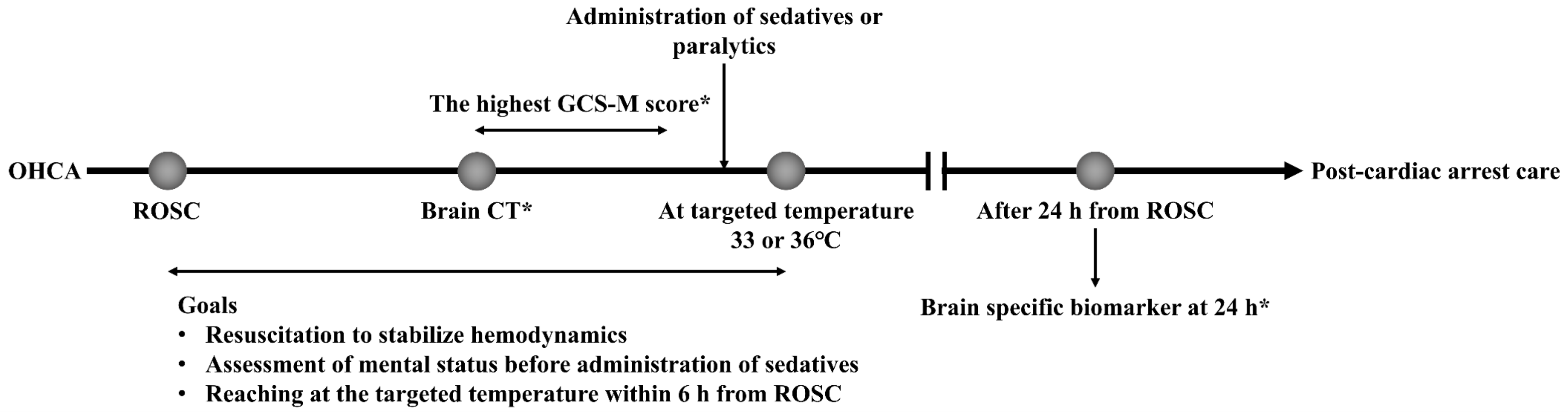

***Abbreviations*:** OHCA, out-of-hospital cardiac arrest; ROSC, return of spontaneous circulation; CT, computed tomography; GCS-M, Glasgow Coma Scale motor score

Supplementary S2. Cerebral Performance Category (CPC) score

The CPC score ranges from one to five: 1, good cerebral performance or slight cerebral disability; 2, moderate disability, independence in activities of daily life; 3, severe disability, dependence on others for daily support; 4, coma or vegetative state; and 5, death or brain death. This was performed through either face-to-face or telephone interviews.

Table S1. Inter-rater reliability analysis of the calculated GWR-BG on brain CT

	**Reviewer 1**	**Reviewer 2**	**ICC**
GWR-BG, mean ± SD	46 (41.8)	53 (48.2)	0.87

Abbreviations: GWR-BG, grey and white matter ratio at basal ganglia level; CT, computed tomography; SD, standard deviation; ICC, intraclass correlation coefficient

References

1. Cho WH. Organ donation in Korea in 2018 and an introduction of the Korea national organ donation system. Korean J Transplant. 2019;33:83-97.
2. Jeong E, Baik S, Park H, Oh J, Lee Y, Lee JM. First organ donation after circulatory death following withdrawal of life-sustaining treatment in Korea: A case report. J Korean Med Sci. 2021;36:e171.
3. Rosner B. Fundamentals of Biostatistics. Cengage Learning; 2015.
